# The public’s voice about healthcare quality regulation policies. A population-based survey

**DOI:** 10.1186/s12913-015-0992-z

**Published:** 2015-08-14

**Authors:** Renée Bouwman, Manja Bomhoff, Judith D. de Jong, Paul Robben, Roland Friele

**Affiliations:** NIVEL, Netherlands Institute for Health Services Research, PO Box 1568, 3500 BN Utrecht, Netherlands; Dutch Healthcare Inspectorate, PO box 2680, 3500 GR Utrecht, Netherlands; Institute of Health Policy and Management (iBMG), Erasmus University Rotterdam, PO box 1738, 3000 DR Rotterdam, Netherlands; TRANZO (Scientific Centre for Care and Welfare), Faculty of Social and Behavioural Sciences, Tilburg University, PO Box 90153, 5000 LE Tilburg, Netherlands

## Abstract

**Background:**

In the wake of various high-profile incidents in a number of countries, regulators of healthcare quality have been criticised for their ‘soft’ approach. In politics, concerns were expressed about public confidence. It was claimed that there are discrepancies between public opinions related to values and the values guiding regulation policies. Although the general public are final clients of regulators’ work, their opinion has only been discussed in research to a limited extent.

The aim of this study is to explore possible discrepancies between public values and opinions and current healthcare quality regulation policies.

**Methods:**

A questionnaire was submitted to 1500 members of the Dutch Healthcare Consumer Panel. Questions were developed around central ideas underlying healthcare quality regulation policies.

**Results:**

The response rate was 58.3 %. The regulator was seen as being more responsible for quality of care than care providers. Patients were rated as having the least responsibility. Similar patterns were observed for the food service industry and the education sector. Complaints by patients’ associations were seen as an important source of information for quality regulation, while fewer respondents trusted information delivered by care providers. However, respondents supported the regulator’s imposition of lighter measures firstly.

**Conclusions:**

There are discrepancies and similarities between public opinion and regulation policies. The discrepancies correspond to fundamental concepts; decentralisation of responsibilities is not what the public wants. There is little confidence in the regulator’s use of information obtained by care providers’ internal monitoring, while a larger role is seen for complaints of patient organisations. This discrepancy seems not to exist regarding the regulator’s approach of imposing measures. A gradual, and often soft approach, is favoured by the majority of the public in spite of the criticism that is voiced in the media regarding this approach. Our study contributes to the limited knowledge of public opinion on government regulation policies. This knowledge is needed in order to effectively assess different approaches to involve the public in regulation policies.

## Background

In the wake of various high-profile incidents such as the Mid Staffordshire NHS Foundation Trust scandal in the United Kingdom, several countries including the Netherlands have faced comparable organisational crises and problems with achieving political goals such as public confidence in healthcare, legitimacy and accountability of regulators in healthcare [1–7]. The criticisms expressed in the media, by politicians and by patient organisations are often directed at the regulators’ cooperating approach in cases where healthcare providers fail to comply with quality standards. Furthermore, it is claimed that regulators fail to respond to patients’ complaints [4, 7].

Although it is often recommended to involve the public as they are the final clients of the regulator’s services [8, 9], their opinions on regulatory policies have only been discussed in research to a limited extent. The main research question in this study is therefore whether there are discrepancies between the values and opinions of the public and the current values of policies and strategies for regulation of healthcare quality, and if so, what are these discrepancies? The Dutch situation is used as a case study.

The next paragraph addresses important theoretical concepts underlying regulation, followed by a description of healthcare quality regulation policies and related issues in the Netherlands. We then explain the methods used in this study, followed by the results and discussion.

### Responsive regulation

Internationally, regulation in various industries such as healthcare, finance and environmental businesses is based on the theory of ‘responsive regulation’ of Ayres and Braithwaite (1992) [10, 11]. The basic idea is that the parties being regulated are considered to be trustworthy and intrinsically motivated by social responsibility. According to this theory, strategies of regulation should be flexible, in synergy with the context of those being regulated, and based on dialogue. Regulation based on trust will improve quality of care more effectively, while regulation based on distrust arguably only leads to more sanctions and therefore more capacity on the part of the regulator and ultimately to higher costs to society [10]. Single regulatory strategies are seldom effective. Weaknesses of one strategy can be complemented by strengths of another. A wide array of strategies such as monitoring performance indicators and targets, incident reporting systems, and more stricter measures as criminal penalties should together contribute to the effectiveness of regulation [10, 12]. Regulatory compliance is encouraged by using cooperation, persuasion, inspection and enforcement notices in the first instance, and secondarily by applying heavier measures in the case of riskier behaviour. This vision is often described as ‘high trust, high penalty’ [10]. This strategy corresponds to the international trend of government functions changing from the old “commanding and controlling” to “steering not rowing”, whereby responsibilities are shifted from the government to the field and new governing mechanisms are introduced such as marketisation of public sectors [4, 13–17]. Another important component of the theory is ‘tripartism’, which is proposed as a mechanism for empowering public interest groups and decreasing the risk of regulatory capture. Furthermore, tripartism can prevent conflicts of values between the different stakeholders. In tripartism, a public interest group participates as a third group in the regulatory process: it is given power by being granted access to all the information that is available to the regulator, and by being offered a seat at the negotiation table for enforcement and compliance [3, 10, 18–21]. In many countries, involvement of the public in regulation is on the policy agenda and different approaches are being considered, such as using the experiences of the public at large [5, 12, 22–24]. However, research has shown that public interest in regulatory agencies is limited, as is the public visibility of these agencies [25–27]. Low public interest may not be a great problem, as these agencies interact primarily with the industry rather than with the general public. However, regulators often do tend to become visible to the public in times of crisis [27, 28]. Scandals and incidents and the accompanying media attention can have a direct influence on the regulators’ reputation [28–30], and may possibly jeopardise public confidence in the industry and its regulation [4, 7, 31]. Although regulation is often defined as “sustained and focused control exercised by a public agency over activities that are valued by a community” [32, 33], research shows that in risk cases involving for instance genetically modified food or radioactive waste, the public does not regard the government regulator as having the same values as themselves [34]. This also implies that it is important for ensuring the legitimacy, public accountability and transparency of a regulator and for involving the public in regulation policies that the values of regulatory policies are consistent with the values of communities. Differences between the values and opinions of the public and the current values of policies and strategies for regulation and underlying ideas of the theory of ‘responsive regulation’, are the main focus of this article.

### Dutch healthcare quality regulation policies

In the Netherlands, the healthcare system was reformed into a regulated market system in 2006 [35]. Before the introduction of this reform, two types of healthcare quality regulation could be distinguished: state regulation and professional self-regulation. Since the competition mechanisms were introduced, the market was supposed to be a new complementary governing mechanism and the state’s function followed a more decentralised approach [17, 35, 36]. In this system, the focus on patient choice and transparency of quality of care has increased [13, 15–17]. Since the introduction of the Quality Act (1996), care providers have been given more responsibilities and are supposed to develop quality standards. The Dutch Healthcare Inspectorate monitors performance against these standards (more information about monitoring and enforcement strategies in Table 1). However, the Netherlands has also seen several high-profile incidents in healthcare that led to concerns in society and a heated political debate about the Inspectorate [18, 36–39]. It was argued that the Inspectorate failed to respond to emerging signals including patients’ complaints and it should have enforced the rules more strictly, because its actions had been too hesitant and trusting of care providers who were not complying with quality standards. Members of the Dutch House of Representatives spoke of “the debate representing the gap between the public and politics, but in miniature”. It was stated that the public and their complaints deserve more attention and should be involved in regulation policies [40]. In the Netherlands, those problems regulators experience are not unique to the healthcare sector. State regulators in the food service industry and education sector face similar incidents and reputational losses [41]. Therefore, this article aims to provide a broader picture of public values and opinions about state agencies and their role in risk regulation.

**Table 1:** Regulation and enforcement instruments of the Dutch Healthcare Inspectorate

In the Netherlands, the Dutch Healthcare Inspectorate is the body appointed by the government to supervise and regulate quality of healthcare. It is an independent part of the Ministry of Health, Welfare and Sports. The Inspectorate pays regular visits, which become more frequent if care providers do not comply with quality standards. Both care providers and the public can report incidents or lodge complaints. However, the Inspectorate’s statutory tasks mean that it cannot handle complaints by individual patients unless the complaints are structural or very severe
Information about the quality of care is collected and analysed to signal potential risks. Information sources include the following:
- System based supervision (monitoring of internal quality systems and governance arrangements)
- Performance indicators
- Reporting of incidents (by the public or care providers)
- Detection of prosecutable facts
- Thematic supervision
The Inspectorate is authorised to use the following regulation and enforcement instruments:
- Advice and incentives (consultation, campaigns);
- Corrective measures (impose improvement plans, strengthened monitoring);
- Administrative measures (command, advice to the Minister to issue a direction, penal sum, administrative fine);
-Measures under criminal or disciplinary law.

## Methods

### Questionnaire

We developed questions reflecting the concepts of the theory of ‘responsive regulation’, ‘high trust, high penalty’, and ‘tripartism’.

Firstly, in order to explore public opinion about the concept of ‘responsive regulation’ and the role and position of the state regulator with respect to the regulated parties and other stakeholders, we developed questions about the responsibilities of professionals, the government and other quality-of-care stakeholders. We included equivalent questions concerning quality regulation in the food service industry and in education, in order to assess whether public opinion is unique to the health sector or if it represents more common attitudes regarding responsibility. The Dutch Inspectorate of Education is part of the Ministry of Education, Culture and Science. The Netherlands Food and Consumer Product Safety Authority is part of the Ministry of Economic Affairs. They also base their regulation policies on the theory of ‘responsive regulation’ [42, 43].

In each sector, seven stakeholders were represented: the state regulators (Dutch Healthcare Inspectorate, Dutch Inspectorate of Education, Netherlands Food and Consumer Product Safety Authority); users (patients, students and their parents, and consumers); executive roles (care providers, teachers, and personnel who prepare food); direct colleagues of the executive roles in the three sectors; managers in the three sectors; ministers (Minister of Health, Welfare and Sports, Minister of Education, Culture and Science, Minister of Economic Affairs); and the European Union. For each stakeholder, respondents were asked to select an answer on a five-point scale, where one meant no responsibility and five meant full responsibility.

The other questions focused mainly on regulation of quality of healthcare by the Dutch Healthcare Inspectorate. The concept of ‘tripartism’ was explored by enquiring about the patients’ responsibility for quality of care and the role patient information should have in monitoring healthcare quality. The questions also included existing information sources used by the Inspectorate, such as complaints from members of the public, complaints from care providers, and quality information supplied by the care providers themselves. In addition, sources for collecting information that are currently not used by the Inspectorate were included, such as searching the Internet for complaints.

Furthermore, the concept ‘high trust, high penalty’ was operationalised into questions focusing on what respondents considered to be good methods for regulating the quality of care. Respondents were asked what sanctions the Inspectorate should impose when care providers fail to provide adequate quality of care. Possible sanctions ranged from soft measures such as ‘double-checking the care institution’ to stricter measures such as ‘closing the care institution’. Possible answers were ‘totally disagree’, ‘disagree’, ‘neither disagree nor agree’, ‘agree’ and ‘totally agree’. The questionnaire was assessed by a permanent committee with delegates from several stakeholder organisations in healthcare such as of the Ministry of Health, Welfare and Sports, the Healthcare Insurers Board, and the Federation of Patients and Consumer Organisations. Their feedback was used to finalize the questionnaire.

### Panel

The questionnaire was submitted in February 2013 to a sample of 1500 members of the Dutch Healthcare Consumer Panel. The Dutch Healthcare Consumer Panel at that time consisted of approximately 6000 people aged 18 and older. A sample of 1500 persons that is representative of the Dutch population was drawn from the Healthcare Consumer Panel. The composition of the sample was compared with the general population in the Netherlands based on data from Statistics Netherlands [44], in order to make it reflect the composition of the Dutch population. Membership of the panel lasts for a maximum of 5 years. Members can quit at any time. New panel members are sampled from the general population and selected on basic characteristics needed to keep the panel representative for the Dutch population. This renewal also ensures that members do not develop specific knowledge of healthcare issues and that questionnaire fatigue does not occur. Questionnaires can be received by post or through the Internet, based on the preference of the member. To increase the response rate, two electronic reminders and one postal reminder were sent to members who had not responded yet. The Dutch Healthcare Consumer Panel is registered with the Dutch Data Protection Authority (no. 1262949) [45].

### Ethics statement

Our study complied with the Helsinki Declaration where applicable. According to the Dutch ‘Medical Research involving human subjects Act’, neither obtaining informed consent nor formal ethical approval for this study was required [46]. No medical interventions were involved and the impact of the questionnaires on daily life was considered minor and thus the welfare and rights of the panel members were protected. Panel members were free to answer the questions or not.

### Statistical analyses

In order to obtain a ranking of responsibility of the seven stakeholders in the three sectors, mean scores for responsibility were calculated. For each sector (healthcare, education and food service) respondents could rate responsibility on a five-point scale for each of the seven stakeholders. Differences between responsibility scores of groups of stakeholders were analysed by creating pair-wise comparisons using the Wilcoxon signed rank test [47].

For the other questions, about information sources and methods of regulation, the first two and last two answer categories were combined. Those results are presented descriptively.

Background characteristics of the study sample were compared to the characteristics of the Dutch population. Data on the Dutch population was obtained from Statistics Netherlands. Research on consumer behaviour shows that younger and more highly educated respondents have more critical attitudes towards services [48]. Therefore, differences in age categories, education levels and the extent to which respondents knew about the Inspectorate were therefore tested by chi-squared tests. This was not possible for ethnicity because almost all respondents were from Dutch origin.

*P*-values of <0.05 were considered to be significant. The data was analysed using the statistical software program STATA version 12.1.

## Results

In total, 875 respondents returned the questionnaire (response rate: 58.3 %). Almost half of the respondents were female (47.7 %). They ranged in age from 18 to 87, with a mean of 51.4. More than half (57.6 %) of the respondents had a medium level of education. The study sample is ethnically less diverse than the overall Dutch population. See Table 2 for the study sample characteristics compared to the characteristics of the general Dutch population. With the exception of educational level and ethnicity, the sample is comparable to the Dutch population.

**Table 2:** Background characteristics of study sample and Dutch population^a^

	Number	Study sample %	Dutch population (18 and older) 2013 %
Gender	875		
Female	416	47.7 %	50.5 %
Male	458	52.3 %	49.5 %
Age	875		
18–39	275	31.4 %	34.0 %
40–64	405	46.3 %	44.8 %
65 and older	195	22.3 %	21.2 %
Ethnicity	874		
Dutch	840	96.1 %	78.9 %
Other	34	3.9 %	21.1 %
Education level	841		
Low (none, primary school or prevocational education)	132	15.7 %	30.4 %^b^
Medium (secondary or vocational education)	484	57.6 %	40.3 %^b^
High (professional higher education or university)	225	26.8 %	28.3 %^b^
Work in healthcare	846		not available
No, I have never worked in healthcare	590	69.7 %	
Yes, I am currently working in healthcare	122	14.4 %	
Yes, I worked in healthcare in the past	134	15.8 %	

^a^Data about the Dutch population come from Statistics Netherlands

^b^These percentages apply to the Dutch population aged 15–65 in 2012. The educational level of the remaining percentage is unknown

The majority (76.3 %) of the respondents of the Dutch Consumer Panel reported some degree of knowledge of the Inspectorate’s work, and about one in ten respondents indicated that they knew exactly what the Inspectorate does. The remaining 14.6 % admitted a lack of knowledge. Additional analysis showed that respondents who are currently working or previously worked in healthcare (30.2 %) were significantly more likely to report knowing, either to some extent or very precisely, what the Inspectorate does.

Respondents rated the Inspectorate to bear most responsibility for the quality of healthcare, assigning it a significantly higher score than care providers (Table 3). Next in ranking came the care providers, the minister, managers, colleagues of care providers, and finally the European Union. Patients were rated to bear the least responsible for quality of healthcare, and this result was statistically significant. The same applies for students and their parents in the educational setting and consumers in the food service industry. The education sector showed approximately the same order of responsibility of stakeholders as healthcare, except for the positions of the managers and the minister being reversed. Significant differences were found between the Dutch Inspectorate of Education and teachers, but not between teachers and managers. In the food service industry, both the personnel who prepared food and the food sector managers were rated as bearing slightly more responsibility than the Netherlands Food and Consumer Product Safety Authority. However, this was not significant.

**Table 3:** Mean scores on responsibility (1 = no responsibility, 5 = full responsibility) of various stakeholders for quality in the Dutch healthcare, education and food service industry (*N* = 819-838)^a^

	Health care	Education	Food service
Dutch Healthcare Inspectorate | Dutch Inspectorate of Education | Netherlands Food and Consumer Product Safety Authority	4.42^a^	4.52^a^	4.36^a^
Care providers | Teachers | Personnel who prepare food	4.29^a^	4.46	4.43
Minister of Health, Welfare and Sports | Minister of Education, Culture and Science | Minister of Economic Affairs	4.17	4.23	3.74^a^
Managers	4.11^a^	4.38^a^	4.41
Direct colleagues of care providers | Direct colleagues of teachers | Direct colleagues of personnel who prepare food	3.92^a^	4.20^a^	4.13^a^
European Union	3.41^a^	3.48^a^	3.32^a^
Patients | Students and their parents | Consumers	2.98	3.22	2.70

^a^Significant score of responsibility compared to group of stakeholders with lower score. Intergroup comparisons were tested using the Wilcoxon signed rank test [47]. *P*-values of <0.05 were considered significant

**Fig. 1:**
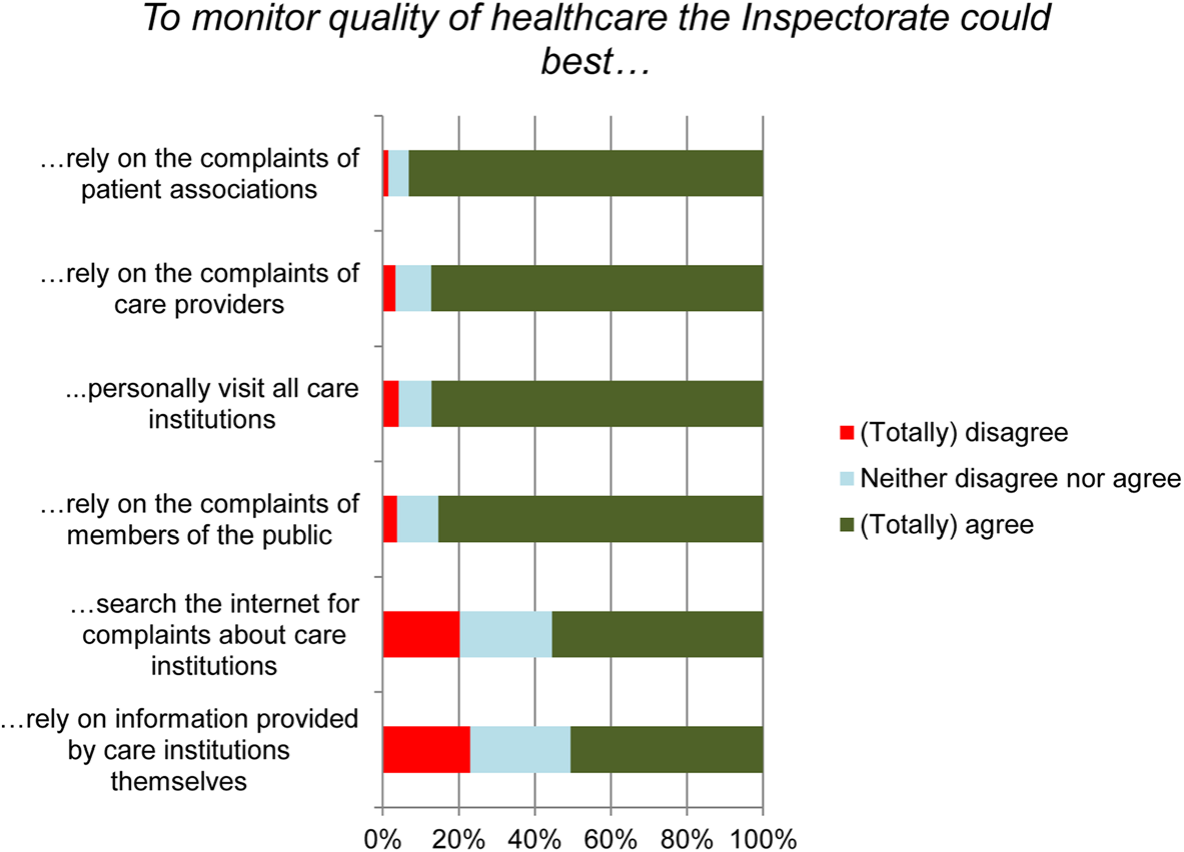
Evaluation of sources for monitoring healthcare quality by the Dutch Healthcare Inspectorate according to respondents of the Dutch Healthcare Consumer Panel (*N* = 818-838)

Respondents were asked what information sources the Inspectorate could best rely on to monitor healthcare quality (Fig. 1). The majority of respondents (93.2 %) agreed (totally or partially) that the Inspectorate could best rely on the complaints of patient associations. In addition, a large majority (87.1 %) agreed (totally or partially) that the Inspectorate should visit all care providers. In addition, the respondents’ opinion was that the Inspectorate should rely on sources such as complaints of care providers (87.3 %) and members of the public (85.3 %). Fewer respondents (approximately half) agreed (totally or partially) that the Inspectorate should rely on information provided by care institutions themselves, whereas 23 % were of the opinion (totally or partially) that it should not.

**Fig. 2:**
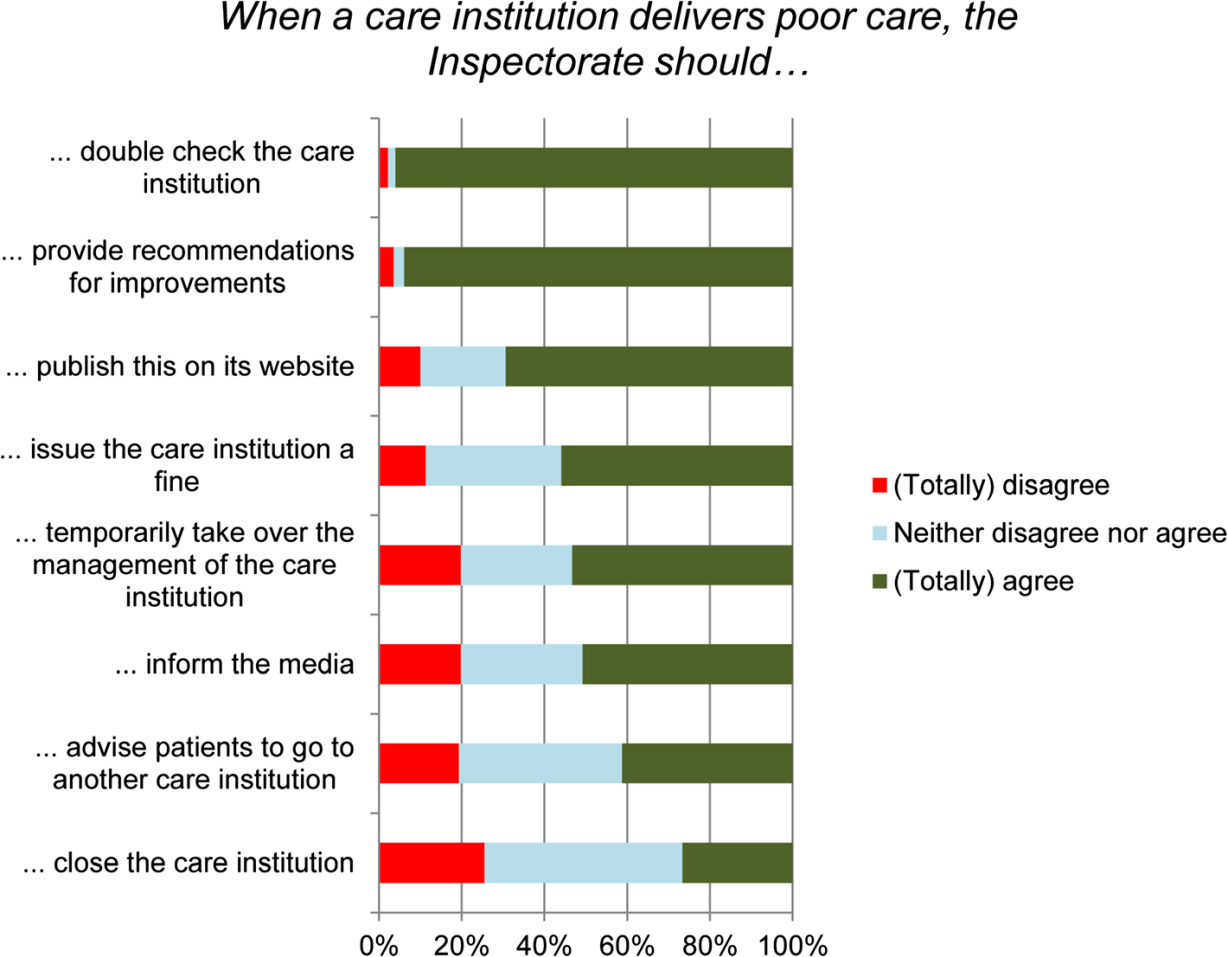
What the Healthcare Inspectorate should do when a care institution delivers poor care according to respondents of the Dutch Healthcare Consumer Panel (*N* = 818-832)

Respondents were asked what measures the Inspectorate should take in cases of poor care (Fig. 2). If a healthcare provider delivers poor care, the majority indicated that the Inspectorate should double-check the care institution (96.4 %) and provide recommendations for improvements (93.9 %). In addition, about 70 % of respondents agreed (totally or partially) that the Inspectorate should publish poor care delivery on its website. With respect to other possible regulatory measures, allowances should be made for the fact that between 20 and 48 % of the respondents answered indifferently (‘neither disagree nor agree’). Slightly more than half of the respondents indicated that the Inspectorate should issue a fine when poor care was provided. Furthermore, 53.3 % of the respondents agreed (totally or partially) that the Inspectorate should temporarily take over the management of a poorly performing care institution. Slightly more than a quarter of all respondents indicated that the healthcare institution should be closed if it provides poor care.

We analysed whether there were differences in the answers given by different age groups, educational levels and knowledge about the Inspectorate. Some significant differences were found. Less highly educated and older respondents tended to agree more often on some questions that the Inspectorate should respond more actively than suggested by respondents in the other categories.

For instance, respondents in the two older age groups and those with a low or medium level of education agreed more often that the Inspectorate should advise patients in cases of poor care delivery to go to another care institution and inform the media than respondents from the other groups did (*p* = 0.000-0.001). Furthermore, less highly educated respondents agreed more often that the Inspectorate should search the Internet for complaints about care providers than respondents from the other groups did (*p* = 0.02). In addition, less highly educated respondents agreed more often that the Healthcare Inspectorate should issue fines in cases of poor care delivery than more highly educated respondents (*p* = 0.03). Lastly, respondents who admitted a lack of knowledge about the Inspectorate’s work, tended to answer indifferently more often on some questions.

## Discussion

This study aimed to explore the opinions and values of the public regarding healthcare quality regulation policies, analysing the Dutch situation as a case study. Similarly to other countries such as the UK, the Netherlands had some high-profile incidents in which the regulator failed to respond to various emerging signals, including patients’ complaints. These led to concerns about public confidence in healthcare and the regulator.

Internationally, political visions on governance and regulation are changing from centralised to decentralised approaches and responsibilities are being shifted from the government to the field [4, 13–17]. In the Netherlands, this changing vision resulted in the introduction of the Quality Act in 1996, which made care providers primarily responsible for the quality of care. In this framework, regulation relies on internal monitoring and self-regulation, on the basis of which the regulator monitors performance [17, 36]. This vision fits with the theory of responsive regulation, in which regulators entrust those being regulated to take their responsibilities [10]. This study shows that the majority of the public partly support this idea: the public assigned a high degree of responsibility to care providers. However, a fundamental discrepancy became apparent: the predominant rhetoric of decentralisation of responsibilities was not supported and the majority of the public seem to have little confidence in the internal monitoring of quality by care providers and the use of this information for regulation. Other studies also found that a large proportion of the public assign responsibility for promoting safety and preventing medical errors in healthcare to state agencies [31, 49]. Moreover, this study shows that there is a generalised idea among the public that the state regulator has a prominent role, as the same patterns were observed for the food service industry and the education sector. Apparently, according to the majority of the public, the internal monitoring of quality and safety of healthcare cannot simply be left to the goodwill of the care providers. Nevertheless, although some differing opinions were found among older and less well-educated respondents, the majority support the regulators’ gradual approaches of imposing measures to care providers who fail to comply with quality standards, as proposed by the theory of responsive regulation [10]. Thus, the majority prefer a greater responsibility and an active role by the regulator with regard to gathering information but not a stricter approach with regard to imposing measures for the state regulator.

It has been stressed in several studies that more democratic approaches to regulation, such as ‘tripartism’, might overcome the conflicts of values that are important to the different stakeholders [3, 10, 18–21, 23]. On the one hand, the majority of the public attach importance to complaints of patient associations as a source of information for regulation. This is an interesting finding, as questions have been raised in several European countries about how patients’ complaints should be valued and have a place in the regulatory process, and public participation in regulation is an important item on the policy agenda [2, 3, 5, 18, 24, 36, 37, 40]. The use of patients’ complaints can be seen as a reduced form of tripartism whereby services become more responsive to and learn from their users. Actually, The Mid Staffordshire Public Inquiry showed that inferences about general patient safety can be gained from individual complaints. Moreover, in this case, individual complaints even indicated dramatic systemic failures [2]. A voice for the patients provides information about ‘blind spots’ that care providers are unaware of; this is also called ‘soft intelligence’ [7]. In this respect, it should be investigated what value complaints could have for regulation of healthcare quality and what those who report complaints to regulators themselves expect from their complaint in the process of healthcare quality regulation. On the other hand, patients were also considered to bear least responsibility for quality of care by the public in this study. This might undermine the goals of the reform of marketisation in healthcare towards more ‘active patient choice’ and more responsibility for patients. Furthermore, the role of patient organisations and their expected role of participating in decision-making processes might be at stake [3, 12, 13, 22]. In addition, it might indicate that the majority of the public do not favour intensive or active methods of ‘tripartism’ in the regulatory process, but instead support more collective forms of participation. This suggestion requires further research, which should include the public’s and patient’s perspectives.

### Strengths and weaknesses

One strength of this study is its large study sample. However, the response rate was moderate which may have caused non-response bias. This sample is comparable to the Dutch population in terms of age and gender, although not with respect to educational level. With respect to the different background variables, we analysed differences in answers. Some significant differences were found in the answers of older and less highly educated respondents. This means that different opinions of subgroups among the public can exist. This should be taken into account when involving the public in regulation policies.

It is striking that a considerable proportion of the respondents answered indifferently. This was also apparent in other studies on public perceptions of the Inspectorate [25, 26]. The regulator might be a ‘low interest good’, its visibility might be low, or respondents might have too little knowledge to answer the questions. However, less than 1 % answered indifferently on all items of Figs. 1 and 2, so this does not mean that the public have no opinions or expectations about healthcare regulation. Furthermore, people might have or might gradually develop more general or common-sense ideas about the Inspectorate and its responsibilities, especially when it attracts media attention. Lastly, it remains unsure whether the same questions about healthcare, food service industry and education sector have equal connotations to the respondents. Therefore, the outcomes with respect to the comparison of the three sectors should be interpreted cautiously.

## Conclusion

Many countries face problems of public accountability, legitimacy and transparency of regulators. To tackles these issues, it is important that the values of regulatory policies are consistent with the values of the public. This study shows that there are discrepancies and similarities between public opinion and regulatory policies. A gradual, and often mild approach with regard to imposing measures to failing care providers, is favoured by the majority of the public in spite of the criticism that is voiced in the media regarding this approach. However, the majority of the public do not support decentralisation of responsibilities of the regulator. This applies not only to healthcare, but also to other industries. Furthermore, the majority agree that the patients’ voice and especially their complaints should play a pivotal role in regulatory policies. Moreover, a form of collective participation by the general public or patients in the regulatory process can potentially overcome the conflict in values between the public and regulatory policies. It also provides information about ‘blind spots’. It would be worthwhile to explore which specific forms of involvement of the public are most suitable while taking into account differing opinions of subgroups, as this would provide a valuable addition to the quality information delivered by healthcare providers. Our study contributes to the limited knowledge of public opinion on government regulation policies. This knowledge is needed in order to effectively assess different approaches to involve the public in regulation policies.
